# Influence of tissue conductivity on foetal exposure to extremely low frequency magnetic fields at 50 Hz using stochastic dosimetry

**DOI:** 10.1371/journal.pone.0192131

**Published:** 2018-02-07

**Authors:** Serena Fiocchi, Emma Chiaramello, Marta Parazzini, Paolo Ravazzani

**Affiliations:** CNR Consiglio Nazionale delle Ricerche, Istituto di Elettronica e di Ingegneria dell’Informazione e delle Telecomunicazioni IEIIT, Milan, Italy; Sudbury Regional Hospital, CANADA

## Abstract

Human exposure to extremely low frequency magnetic fields (ELF-MF) at 50 Hz is still a topic of great interest due to the possible correlation with childhood leukaemia. The estimation of induced electric fields in human tissues exposed to electromagnetic fields (EMFs) strictly depends on several variables which include the dielectric properties of the tissues. In this paper, the influence of the conductivity assignment to foetal tissues at different gestational ages on the estimation of the induced electric field due to ELF-MF exposure at 50 Hz has been quantified by means of a stochastic approach using polynomial chaos theory. The range of variation in conductivity values for each foetal tissue at each stage of pregnancy have been defined through three empirical approaches and the induced electric field in each tissue has been modelled through stochastic dosimetry. The main results suggest that both the peak and median induced electric fields in foetal fat vary by more than 8% at all gestational ages. On the contrary, the electric field induced in foetal brain does not seem to be significantly affected by conductivity data changes. The maximum exposure levels, in terms of the induced electric field found in each specific tissue, were found to be significantly below the basic restrictions indicated in the ICNIRP Guidelines, 2010.

## Introduction

The potential susceptibility of foetuses to exposure to electromagnetic fields (EMFs), especially during organogenesis, together with their possible higher sensitivity to EMF makes the assessment of foetal exposure levels a topic of high interest [1–3]. Therefore, the scientific community [4,5] has lately claimed it is necessary to assess foetal exposure to extremely low frequency magnetic fields (ELF-MFs) at 50 Hz. Epidemiological studies have indeed indicated a possible correlation between exposure to ELF-MF and human cancer, especially childhood leukaemia [6,7]. ELF-MF exposure could be a possible trigger for that disease, whose first insult could arise during prenatal life [8]. However, no biological mechanism is currently able to explain a causal relationship between ELF-MF exposure and children leukaemia [9] and therefore further efforts are required to inform the whole health risk assessment process.

So far, the studies that have analysed foetal ELF-MF exposure risk by estimating the strength of the induced electric field and the current density in foetuses [10–13] have used deterministic dosimetry based approaches and very simplified foetal models and few tissues (soft tissues, brain, and skeleton). More recently, Liorni and colleagues [14] performed a detailed analysis of the foetal exposure in each specific tissue at 3, 7, and 9 months of gestational age (GA), by means of advanced high resolution voxel pregnant woman’s models in which 15, 17 and 26 foetal tissues were distinguished at each stage of pregnancy [15].

Sinc the variability of EMF exposure in real exposure scenarios depends on both the exposure configuration (i.e. the source types, the frequency and the polarization of the incident EM field) and the physical characteristics of the subjects being exposed (i.e. morphology, dielectric properties and postures), Liorni and colleagues also estimated the variation in foetal exposure as a function of the more likely foetal postures at each stage of pregnancy [14] and as a function of the incident magnetic field orientation in 3D-space [16]. In order to reduce the computational cost necessary to assess all of these different exposure configurations, the authors adopted a stochastic approach based on polynomial chaos theory [17][18] to build surrogate models of the induced electric field in each tissue as a function of the magnetic field orientation.

As stated above, the variability in the induced field by EMF exposure is also strictly linked to the dielectric tissue properties, which change both across subjects of the same age and with respect to the adult values. Indeed, there is evidence that dielectric tissue properties decrease with age and, in some foetal tissues, are extremely different form the adult values [19–22]. However, measurements of foetal dielectric tissue properties are scarce [23–26] and all have been performed in the radiofrequency (RF) and microwave ranges.

Two previous studies [10,11] that have analysed the variation in foetal exposure to ELF-MF have taken into account the variation in dielectric tissue properties and have adopted empirical approaches to estimate the increase in the conductivity (which is the only dielectric property that must be accounted for in the low frequency range) of the foetal tissues. The increase in conductivity was obtained by estimating the change in the fraction of water in the foetal tissues with respect to adult tissues [10]. This estimate has been performed based on the approach introduced by Smith and Foster [27], who compared the conductivities as a function of water content in the tissues. Cech and colleagues [11] assigned the conductivities of foetal soft tissues as 10% greater than the average properties of human tissue at 50 Hz.

Starting with these two different empirical approaches, we have defined specific ranges in foetal conductivity variations at 50 Hz, with the purpose of estimating induced electric fields in response to all possible exposure conditions as determined by their uncertainty assignment to assess the foetal exposure variability.

This analysis was carried out following the same approach we adopted in our previous paper [16] by means of the polynomial chaos theory to build surrogate models of the induced electric field in foetal tissues.

## Material and methods

### Polynomial chaos theory to assess ELF-MF exposure

In this study polynomial chaos (PC) theory has been applied to assess foetal ELF-MF exposure by changing the values assigned to foetal tissue conductivity at 3, 7 and 9 months GA, following the PC approach previously described in [16]. Briefly, PC is a spectral method and consists in the approximation of the system output, Y, on a suitable finite-dimensional basis **Ψ**(**X**) of orthogonal polynomials [17,18,28]. For computational purposes, the polynomial expansion is truncated after P terms:

$$Y=M(X)\approx \sum_{0}^{P-1}a_{j}\psi_{j}(X)$$ (1)

where **X** is the random input vector of the input parameters x_i_, *ψ*_j_ are the polynomials belonging to the basis **Ψ** (**X**) and a_j_ are the unknown coefficients to estimate.

In this study the set of input parameters, which are the conductivity values in specific foetal tissues (see details below), are presumed to be uniformly distributed, given that we have no indication about the most probable value to be attributed to them. Therefore, the polynomials, *ψ*_*j*_*(****X****)*, belonging to the basis, **Ψ**(**X**), are made of a family of Legendre polynomials [28].

The coefficients, a_j_, of the PC expansion (1) are estimated by the Least Angle Regression (LAR) algorithm [29,30]. The LAR algorithm collects a series of PC expansions, selecting the most suitable polynomials from the basis, **Ψ**(**X**). The estimates for the coefficients, a_j_, in each PC model built by LAR are then obtained by least-square regression with respect to a series of N observations, **Y**_**o**_ = {y_o_^(1)^, y_o_^(2)^,…,y_o_^(N)^}, of the system output, Y. In this study, **Y**_**o**_ is calculated by using deterministic dosimetry with respect to the experimental design, **X**_**o**_ = {**x**_o_^(1)^, **x**_o_^(2)^,…,**x**_o_^(N)^}, where **x**_o_^(i)^ are the values assigned to the input parameters used in the deterministic dosimetry. The best PC expansion among the series collected by LAR was finally chosen through a leave-one-out (LOO) cross-validation [31, 32], using the corrected relative LOO error *ε*_LOO_ described in [30]. The goodness of the model thus obtained was evaluated through the following expression of relative error:

$$Relative\;Error=\sum_{i=1}^{N}\frac{{\left(Y_{0}(i)-Y(i)\right)}^{2}}{N*var(Y)}$$ (2)

where var(Y) is the best PC expansion variance and the other terms are defined above. For a more detailed discussion of the application of PC theory to ELF-MF exposure refer to [14].

In this study, the system outputs Y modelled by PC expansion are the 50^th^ and the 99^th^ percentile value of root mean square of the foetal tissue-specific induced electric field (respectively indicated as E_50th_ and E_99th_) averaged on a 2 mm side cube. This metric is adopted from the 2010 International Commission of Non-Ionising Radiation Protection Guidelines [33] as the relevant tissue-specific value to be compared with the basic restrictions. However, it should be noted that previous ICNIRP guidelines [34] indicated the current density, **J**, as the relevant quantity that directly affects the electrical excitable structures of Central Nervous System (CNS) tissues. Given that J is linked to electric field, **E**, by Ohm’s Law by the proportionality factor σ, (i.e. conductivity), results based on that metric could be in principle even more affected by conductivity variations.

### Input vector X: Foetal tissues conductivity

The input vector, **X**, of the input parameters is made up of the conductivity values for the foetal brain, bone, fat, skin and spinal cord. Those tissues were chosen because, according to Peyman et al. [20], they present with a systematic variation in dielectric properties that change with age. They represent 52%, 68% and 51% of the total 3, 7, and 9-month foetal volume, respectively.

In this study, the bounds of the range of variation in the conductivity of the selected tissues were identified on the basis of the values of adult tissues [35–38] modified using the three approaches described below. However, it must be noted that these approaches represent only a starting point to determine the variability of the tissue conductivity ranges: they do not have a physiological meaning nor have they been derived by a direct measurement of foetal tissues. As a consequence, they could mimic the actual values for some tissues, but on the other hand could also be affected by additional approximations, whose degree of quantification is out of the scope of this study.

- According to Cech and colleagues [11] the conductivities of the selected foetal tissues have been estimated by increasing the corresponding adult conductivity values by 50%.
- The second approach is similar to the one adopted by Dimbylow [10], in which the effect of increased conductivity values in the foetus is evaluated by estimating the change in conductivity due to the changes in tissue water content. This approach based on the hypothesis that at low frequencies and for low current densities, cells are poorly conducting compared to the surrounding electrolyte environment and, therefore, the currents circumvent the cells. For this reason tissues and organs can be modelled as a suspension of non-conducting particles surrounded by interstitial fluid, and the Maxwell-Friecke mixture theory can be applied [27]. In this theory, cells are considered as spheres of volume fraction *f*_*v*_ in a continuous medium. Under the hypothesis that cells conductivity is much lower than the surrounding medium (σ_m_), the conductivity of the mixture (i.e. the tissue) σ is expressed as:

$$\sigma ∼2\sigma_{m}(1-f_{v})/(2+f_{v})$$ (3)

According to Dimbylow [10], σ_m_ does not change between foetal and adult tissues, then the tissue foetal conductivity, σ_F_, can be expressed with respect to the adult conductivity, σ_A_, as:

$$\frac{\sigma_{F}}{\sigma_{A}}=\frac{\left(1-f_{vF})(2+{f_{v}}_{A}\right)}{\left(1-{f_{v}}_{A})(2+f_{vF}\right)}$$ (4)

where *f*_*vF*_ and *f*_*vA*_ are the volume fractions of cells in the foetal and adult tissues, respectively.

- In the third approach based on the paper by Peters and colleagues [39] the cells are considered as elongated homogeneously-distributed and randomly-orientated non-conducting spheroids. The estimated conductivity is limited by the bounds:

$$\sigma_{m}{\left(1-f_{v}\right)}^{5/3}\leq \sigma \leq \sigma_{m}{\left(1-f_{v}\right)}^{3/2}$$ (5)

In our study the lower and upper bounds of the foetal conductivity σ_F_ are expressed as a function of the adult conductivity σ_A_:

$$\frac{\sigma_{F}}{\sigma_{A}}={\left(\frac{\left(1-f_{vF}\right)}{\left(1-{f_{v}}_{A}\right)}\right)}^{s},\;s=\frac{5}{3},\frac{3}{2}$$ (6)

A review of the published literature about foetal and adult body composition [40–44] has been performed to find the fraction of water in the tissues selected for our study. In Table 1 the fraction of water *f*_*W*_ in the selected foetal tissues and in the corresponding adult tissues are indicated. The volume fraction, *f*_*v*_, is the complementary fraction of *f*_*w*_.

**Table 1:** Fraction of water for the tissues selected for the analysis in the foetus at 3, 7 and 9 months GA and in the adult.

Foetal Tissue	Fraction of water *(f*_*w*_*)*			
	3 mGA	7 mGA	9 mGA	Adult
Brain	0.932[42]	0.920[43]	0.900[43]	0.755[44]
Bone	0.412[42]	0.295[42]	0.210[42]	0.122[44]
Fat	0.940[41]	0.850[41]	0.830[41]	0.210[44]
Skin	0.920[42]	0.900[43]	0.830[43]	0.653[44]
Spinal Cord	0.932[42]	0.920 [43]	0.900[43]	0.750[40]

The conductivity values of the selected foetal tissues obtained by applying the three approaches previously described are summarized in Table 2, which also shows the corresponding adult conductivities [35–38].

**Table 2:** Conductivity (S/m) values, calculated following the three approaches for 3, 7, and 9 months GA compared to the adult values.

Foetal Tissue	Conductivity [S m^-1^]				
	1^st^ approach [σ_A_+50%]	2^nd^ approach [Eq (3)]	3^rd^ approach [Eq (5)]		Adult
			s = 5/3	s = 3/2	
3 mGA					
Brain	0.278	0.248	0.263	0.253	0.185[38]
Bone	0.143	0.357	0.722	0.590	0.095[38]
Fat	0.117	0.472	0.946	0.737	0.078[37]
Skin	0.150	0.159	0.177	0.167	0.100[35]
Spinal Cord	0.041	0.036	0.039	0.037	0.027[36]
7 mGA					
Brain	0.278	0.243	0.257	0.249	0.185[38]
Bone	0.143	0.244	0.414	0.357	0.095[38]
Fat	0.117	0.409	0.800	0.634	0.078[37]
Skin	0.150	0.154	0.171	0.162	0.100[35]
Spinal Cord	0.041	0.036	0.038	0.037	0.027[36]
9 mGA					
Brain	0.278	0.236	0.248	0.241	0.185[38]
Bone	0.143	0.169	0.235	0.215	0.095[38]
Fat	0.117	0.396	0.769	0.612	0.078[37]
Skin	0.150	0.137	0.149	0.143	0.100[35]
Spinal Cord	0.041	0.035	0.037	0.035	0.027[36]

The range of variability of the conductivity for each selected tissue at 3, 7, and 9 months GA was defined by choosing the minimum and the maximum of the values reported in Table 2, and is summarized in Table 3. The lower bound of the interval corresponds to the adult conductivity value for all tissues, whereas the upper bound varies accordingly to the maximum determined using the different three approaches. It is noteworthy that this variability range should be large enough to encompass the possible physiological variability range and also to include errors due to dielectric property measurement [45].

**Table 3:** Range of variability of the input parameters (σ) and percentage increase of the upper bound with respect to the lower bound.

Foetal Tissue	3 mGA		7 mGA		9 mGA	
	Range σ (S/m)	Increase (%)	Range σ (S/m)	Increase (%)	Range σ (S/m)	Increase (%)
Brain	[0.185; 0.278]	50	[0.185; 0.278]	50	[0.185; 0.278]	50
Bone	[0.095; 0.722]	660	[0.095; 0.414]	336	[0.095; 0.350]	268
Fat	[0.078; 0.946]	1113	[0.078; 0.800]	926	[0.078; 0.769]	227
Skin	[0.100; 0.177]	77	[0.100; 0.171]	71	[0.100; 0.150]	50
Spinal Cord	[0.027; 0.041]	50	[0.027; 0.041]	50	[0.027; 0.041]	50

Starting from these conductivity bounds, for each foetal tissue at 3, 7, and 9 months GA, a probability uniform distribution was built and used to define the input range to build the PC expansion to determine the E_50th_ and E_99th_ induced in each foetal tissue.

### Observations Y_o_

As previously mentioned in section (2.1), N observations of the E_99th_ and E_50th_ induced in each of the foetal tissues whose conductivity value is changed, need to be estimated to build the PC expansion using the LAR algorithm. In this study, these observations have been obtained using deterministic dosimetry. The experimental design **X**_**o**_ was generated using a Quasi Monte-Carlo method based on the Sobol function applied on the joint probability density function (PDF) f_X_ of the input parameters [30].

**Fig 1:**
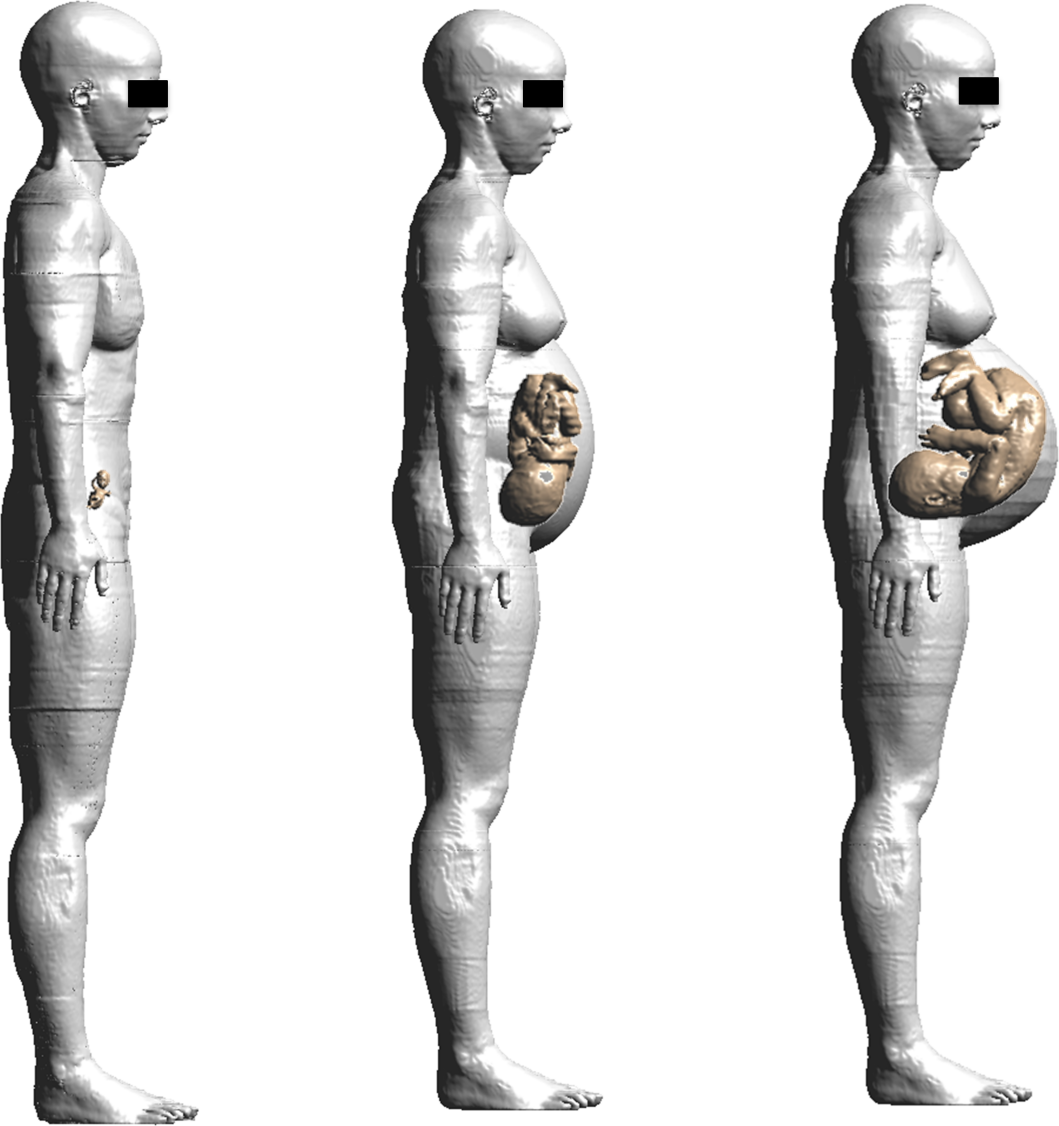
Pregnant women anatomical models. Pregnant woman models at 3, 7 and 9 months GA (from left to right side) were used for the estimation of the observations **Y**_**o**_ by deterministic dosimetry. In each model, the fetus has been highlighted to show its posture in the pregnant woman’s womb.

The electromagnetic simulation procedure adopted in this study follows the one previously used by the authors in [14]. Three high-resolution pregnant woman models at 3, 7 and 9 months GA (Fig 1), based on the model “Ella” of the Virtual Family [46] were used. More detail about the construction of these models is provided in [15].

Simulations were conducted using the Magneto Quasi-Static low frequency solver implemented in the simulation platform SEMCAD X v. 14.8 [47]. The pregnant woman’s tissues (including the foetal tissues) at 7 and 9 months GA were discretized with a grid resolution of 1 mm, whereas at 3 months GA, a grid resolution of 0.3 mm was chosen to allow for thin foetal skin discretization. The conductivities of the woman tissues were assigned according to the data available in literature [35–38]. Details of the chosen conductivity values are described in [14]. The conductivity values for the foetal tissues, whose conductivity values are not supposed to vary, were assigned the same values as the adult tissues.

The pregnant models were exposed to a perfectly homogeneous **B**-field at 50 Hz at 1 μT of amplitude. On the basis of the results already achieved by the authors in [14], the **B**-field orientation with respect to the pregnant woman’s body which induced the highest E_99th_ in the foetal whole-body at each stage of GA was chosen, i.e. the frontal exposure at 3 and 9 months GA and the lateral exposure at 7 months GA.

The PC expansions of E_99th_ and E_50th_ in each foetal tissue were built with N = 20 observations for each GA. This number of observations was chosen as a practical compromise between a mild computational cost and a low relative error, a priori fixed to equal 5%. It indeed permits the construction of accurate surrogate models of E_50th_ and E_99th_ with a relative error, as calculated in (2), which was always lower than 2%.

### Analysis of the foetal exposure

The PC models thus obtained have then been used to generate 10.000 E_50th_ and E_99th_ values, corresponding to the input samples randomly taken within each range estimated for the foetal tissues conductivity (presented in Table 3), and a statistical analysis has been performed to estimate the trend of E_50th_ and E_99th_.

In detail, the E_50th_ and E_99th_ distributions for each tissue of each foetal model derived from the descriptive statistic in terms of minimum, maximum, and quartiles (first quartile Q_1_, median Q_2_, third quartile Q_3_) were evaluated.

In order to further characterize the distributions, the Quartile Coefficients of Dispersion:

$$QCD=\frac{Q_{3}-Q_{1}}{Q_{3}+Q_{1}}$$ (7)

and the Pearson median skewness index:

$$Skewness=3*\frac{Mean-Q_{2}}{StD}$$ (8)

(where “Mean” and “StD” are the mean and the standard deviation of the distributions, respectively) were calculated.

Moreover, we performed a global sensitivity analysis to assess to what extent the variability in each single input parameter affects the output. In detail, we used the variance-based method introduced by Sobol [48],[49], to calculate the Sobol indices, defined as the ratios between the partial variances referred to each input parameter and the total variance of the system output, normalized with respect to the sum of all of the Sobol indices under consideration.

Furthermore, the maximum levels of E_99th_ found in each tissue were compared to the basic restrictions provided by the ICNIRP guidelines for general exposure [33], when the incident magnetic field is set to the reference levels of 200 μT at 50 Hz.

## Results

**Fig 2:**
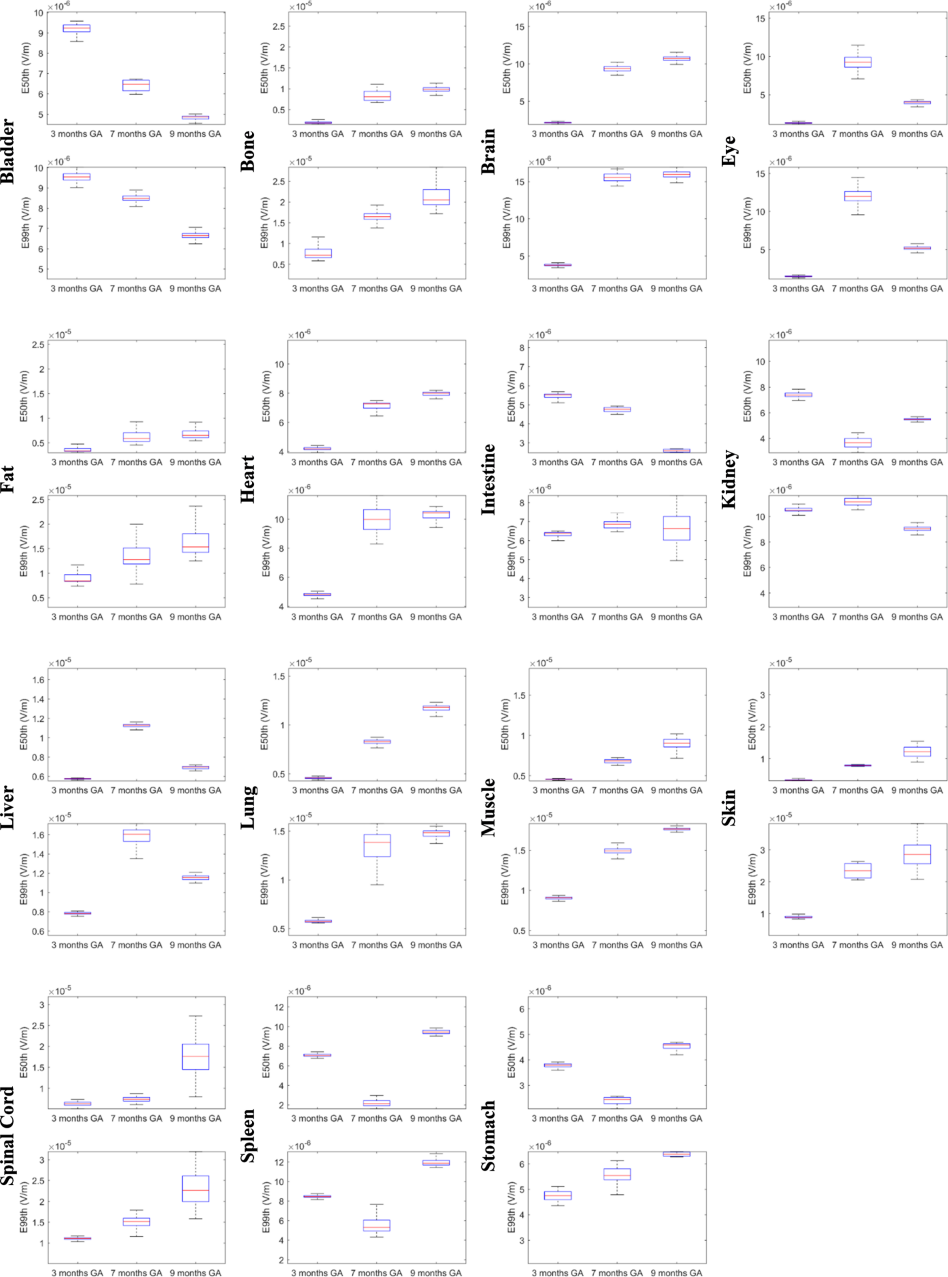
E_50th_ and E_99th_ distributions. Statistic descriptive of E_50th_ and E_99th_ (top and down row) in each foetal tissue at 3, 7, and 9 months GA exposed to a perfectly homogeneous B = 1 μT. The lower and upper bound of the box represent the 25^th^ and 75^th^ percentiles, the line is the median value, and the whiskers are the minimum and maximum values. Scales are adapted to different limits for the sake of readability.

**Fig 3:**
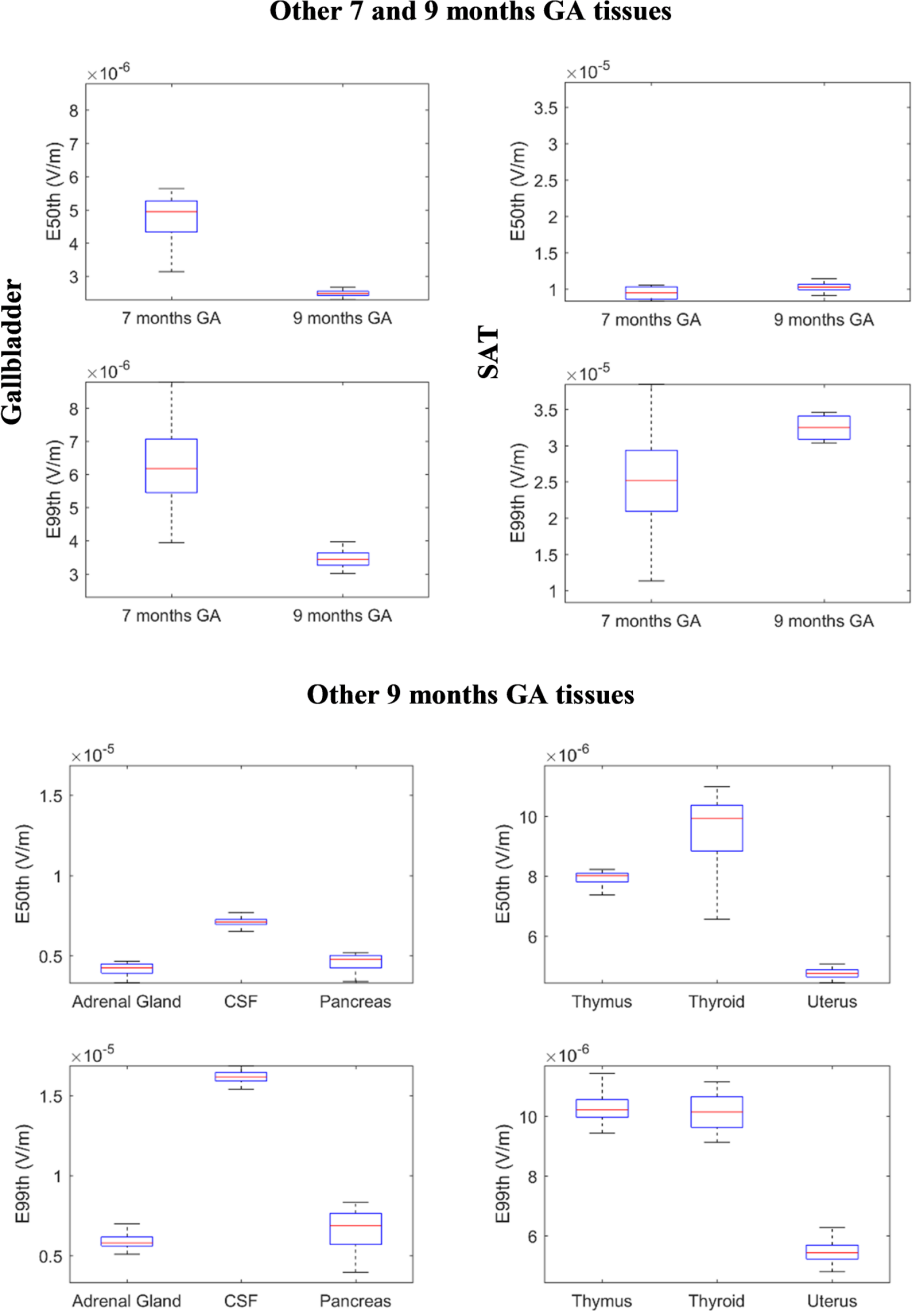
E_50th_ and E_99th_ distributions 7 and 9 months GA tissues. Statistic descriptive of E_50th_ and E_99th_ (in the remaining (from Fig 2) tissues at 7 and 9 months GA.

Fig 2 represents the descriptive statistic as a box-plot (the lower and upper bound of the box represent the first and the third quartiles, the line is the median value and the whiskers are the minimum and maximum value) of the distribution of the 10.000 values of E_50th_ and E_99th_ (top and down row, respectively) in each foetal tissue distinguishable at each stage of pregnancy and estimated through the designated PC models. Similarly, Fig 3 shows the same statistic for the remaining tissues which were not distinguishable in the 3 month GA pregnant woman model.

At 3 months GA the maximum variation in exposure due to the change in the foetal tissue conductivities, expressed as QCD, of the E_50th_ distribution has been found in bone, where it is close to 15%. The tissues which present with the highest variability in E_50th_ are the tissues whose conductivities have been changed (brain, fat, skin and spinal cord) and the eye, whose QCDs are higher than 4%. The variability of the other tissues is lower than 2%.

A more complex exposure level distribution was obtained in the 7 months GA foetus, where the E_50th_ varies the most in the bone and fat (>13%), but also varies considerably in the spleen (12%), varies partially in the eye, gallbladder, heart, kidney, SAT and spinal cord (5–10%), whereas it has a low variation (2–5%) in the bladder, brain, intestine, muscle, and stomach, and a negligible QCD (< 2%) in other tissues.

Similarly, at 9 months GA, the variation in E_50th_ due to the change in the foetal tissues conductivity range between 10 and 17% in the fat, skin and spinal cord, is over 5% in adrenal gland, muscle, pancreas, and thyroid, varies partially (2–5%) in the brain, bone, CSF, eye, gallbladder, intestine, SAT and uterus, and has a negligible variation (< 2%) in all other tissues.

As for the E_99th_ distribution, at 3 months GA, the maximum QCD due to the change in the foetal tissue conductivity, is again in the bone and fat, where it is of up to 13%, whereas it is lower than 5% for all other tissues.

At 7 months GA, the QCD increases up to 16% in the SAT and up to 12–13% in the fat and gallbladder, whereas it ranges between 5–10% in the eye, heart, lung, skin, spinal cord and spleen, and it varies by less than 5% in all other tissues. Similarly, at 9 months GA, the QCD ranges between 10 and 15% in the fat, pancreas, skin and spinal cord, between 5–10% in the bone, gallbladder, intestine and thyroid and varies by less than 5% in all other tissues.

The analysis of the symmetry of both the E_50th_ and E_99th_ distributions, calculated through the Pearson median skewness, reveals that they are, on average, nearly symmetric (i.e. the skewness index is close to zero), but there are some exceptions across the tissues. More specifically, as summarized in Table 4, the fat and bone at all GAs and skin at 3 months GA show strong positively skewed distributions for both of the metrics analysed, whereas intestine at 3 months GA, lung at 7 months GA and heart, lung and pancreas distributions at 9 months GA are strongly negatively skewed.

**Table 4:** Summary of the tissues grouped according to the skewness characteristics of the E_50th_ and E_99th_ distributions.

Skewness	3 months GA	7 months GA	9 months GA
Negatively skewed	Bladder, Intestine	Lung	Heart, Lung, Pancreas
Positively skewed	Fat, Bone, Skin	Fat, Bone	Fat, Bone
Nearly symmetric	All other tissues	All other tissues	All other tissues

**Fig 4:**
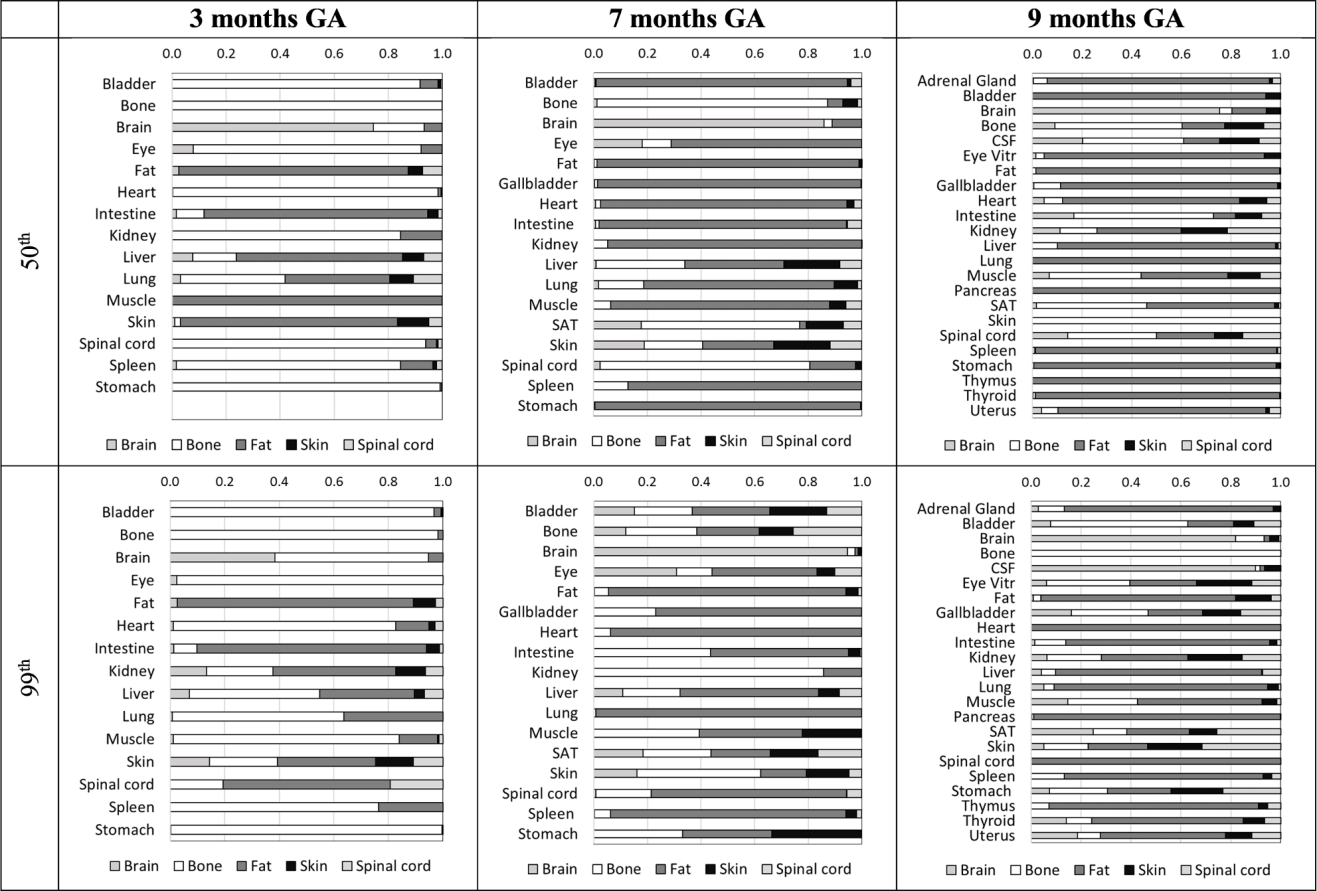
Sobol sensitivity analysis. Normalized Sobol indexes calculated for each tissue, for both the two metric (E_50th_ and E_99th_) analysed at each GA.

Fig 4 shows the results of sensitivity analysis: the normalized Sobol indexes show a similar behaviour in the two metric (E_50th_ and E_99th_) analysed at each GA, suggesting that bone and fat conductivities mainly affect the resulting electric field quantities in all the other tissues. Visual inspection shows that there is a clear prevalence of normalized Sobol indexes referred to fat and bone not only in themselves and in their close tissues, but also in brain, spinal cord and skin.

**Fig 5:**
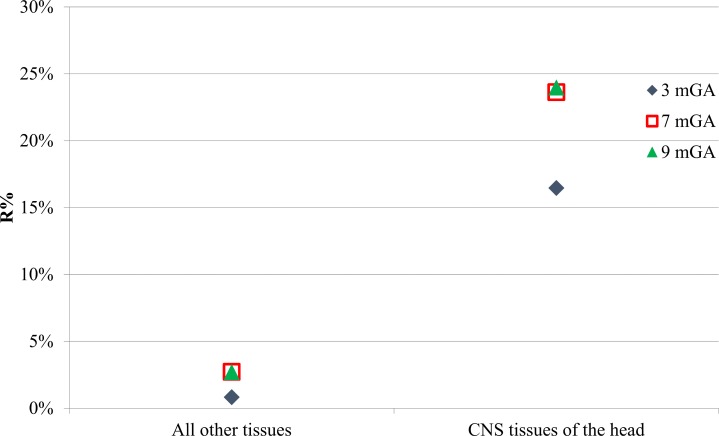
Compliance with ICNIRP guidelines. Percentage Ratio (R%) between the maximum E_99th_ induced in the CNS tissue of the head or in other tissues of the head and body and the corresponding basic restrictions limits made by the ICNIRP Guidelines [33] for general public exposure at each GA.

The maximum E_99th_ values (top whiskers of Figs 2 and 3) achieved in each tissue and at each GA have been compared to the Basic Restrictions made by the ICNIRP Guidelines [33] for general public exposure, i.e. 0.02 V/m for the CNS tissue of the head and 0.4 V/m for all other tissues of the head and the body, considering an exposure to a magnetic field amplitude of 200 μT. Practically, the maximum for exposure to the CNS tissue of the head (corresponding to foetal brain) and for all other tissues of the head and the body (corresponding to fat, SAT, and skin for 3, 7, and 9 months GA, respectively) was multiplied by 200 and then the percentage ratio (R%) with respect to the corresponding basic restriction level was calculated and represented in Fig 5.

From Fig 5, it can be observed that the maximum levels estimated for the electric field induced over all tissues and for all the stages of GAs are substantially less than (< 25%) the maximum levels allowed by the ICNIRP Guidelines for the general public exposure [33].

## Discussion and conclusions

The assignment of dielectric properties is a largely debated issue in computational dosimetry. This is due to: the scarce availability of uniform measures; the uncertainty related to the measurement setup itself (related in turn to probe sensitivity, the conditions and the natural inhomogeneity of the sample, and the repeatability of the measurements, etc.) especially in samples exposed to low frequency fields; the un-reliable correspondence between measures made on animals tissues with the corresponding human tissue; the non-linear behaviour of dielectric properties with respect to exposure frequency; and the systematic variation in the properties of tissue following physiological and pathological changes [44].

With regard to this last issue, some recent studies [10,19–22,50,51] have triggered a question regarding the impact of variation in the dielectric data as a function of age in dosimetry estimates in children, highlighting the possibility that these effects can impact the levels of the metric that are used to quantify the exposure (electric field, current density, Specific Absorption Rate SAR). However, given the high computational cost related to deterministic analysis of the exposure metric effect of any variation in the tissue parameters, previous studies have provided a limited picture of the possible dosimetric outcomes.

In this paper, we have tried to overcome this limitation, by estimating the influence of the conductivity values assigned to foetal tissues and organs in high resolution numerical models used to quantify tissue-specific induced electric fields due to exposure to 50 Hz ELF-MF by means of the stochastic dosimetry. In detail, polynomial chaos theory has been applied to build surrogate models of the induced electric fields in foetal tissues where potential variations in the conductivity of the tissues (brain, bone, fat, skin and spinal cord) were included to calculate deterministic dosimetry. The variation in the conductivity in these foetal tissues was defined using empirical approaches, since conductivity measurements are not yet available in response to ELF-MF exposure at 50 Hz.

Our results show that foetal fat shows the highest level of variation in conductivity with respect to the adult value (Table 3). This results in the largest variation in the median (E_50th_) and the peak (E_99th_) for induced electric field in foetal fat, which was always higher than 8% in terms of the Quartile Coefficient of Dispersion, across all GAs.

In contrast, the foetal brain shows a lower variation in both the median and peak values for the induced electric field of not more than 3% across all GAs. This could be due to the lower range of variability in conductivity for foetal brain (50% maximum difference between the upper and the lower bound of the conductivity range), and to its relative position within the foetal body.

There are tissues, such as the spinal cord, which show relatively little variation in conductivity, but show a considerable variability in induced electric field (Figs 2 and 3), in particular in the 9 month GA foetal tissue (QCD > 13% for both E_50th_ and E_99th_). This discrepancy for the spinal cord could be due the fact that it is completely enclosed in bony tissue, and therefore, at the interface, it is influenced both by the variations in the conductivity properties of the CNS tissues and the variation in conductivity of the bony tissue.

Similarly, there are tissues whose conductivity did not show very much variation, but whose induced electric field levels showed considerably change (Fig 2). This confirms that the relative position of tissues whose conductivity values change with respect to tissues whose conductivity values do not change produce a complex and unpredictable induced electric field distribution.

For the same reason, differences in the variation of induced electric fields across GAs are likely due to differences in the anatomy of the tissues and organs, their position with respect to the surrounding organs, and also the interaction with the incident magnetic field orientation. That is reinforced by the analysis of Sobol indexes, which show that fat and bone conductivities strongly guide the resulting variability in induced electric field in all tissues (Fig 4).

The analysis of the skewness in both the E_50th_ and E_99th_ distributions seems to suggest that variability in conductivity input parameters results in a symmetrical distribution for small tissues (brain and spinal cord) and in a weak positively skewed distribution for extended tissues (in particular bone and fat). Moreover, the induced E_50th_ and E_99th_ distributions for tissues whose conductivity does not change are nearly symmetric. This asymmetry, is also observed in samples derived by deterministic dosimetry, and could be due to the different contributions made by the conductivity of the tissues in the complex induced electric field distribution as a result of magnetic field exposure. In particular, tissues which have a greater increase in conductivity (from Table 3, bone and fat) tend to have a lower induced electric field as a result of the effects of boundary conditions at the interface with adjacent tissues. This results in a distribution in the induced electric field that is more skewed towards small electric field values (positively skewed distribution). Moreover, the behaviour of the Sobol indexes summarized in Fig 4 showed that variability in bone and fat conductivities are the most influential on their own induced electric field results distributions.

In terms of absolute values, the median and maximum levels of induced electric field reported in this study, agree with the ones found in previous studies [14,16], where a decrease in both peak- and median- induced electric fields going into the inner layers, could be expected. However, the expected decrease in the induced electric field in tissues where the conductivity is increased, as occurs when passing from adult conductivities [14] to the mixture theory conductivities (present study), cannot be guaranteed. As a consequence, this data has been used to re-evaluate compliance with the exposure guidelines limits set by ICNIRP [33] (Fig 5).

The analysis shows that, despite factors which cause variations in induced electric fields including changes in tissue conductivities (this study) and magnetic field orientations [16], the foetal exposure to a 50 Hz homogeneous magnetic field of 200 μT amplitude is well within the limits provided by the guidelines (ICNIRP 2010) for general public exposure. Specifically, the reported levels of induced electric field are very close to the ones reported in the only papers which addressed 50 Hz magnetic field exposure of women at different gestational ages [10,14,16], thus reconfirming the same safety considerations previously advanced.

To conclude, this study confirms that stochastic dosimetry is a quick, robust and valuable method to investigate the variability of foetal exposure as a result of the possible uncertainty in the assignment of the foetal tissues conductivity. However, the results suggest that, despite the considerable range of variability in the conductivity values, the effects on the compliance with the exposure guidelines are negligible, whereas we found a limited influence on electric field levels induced in the single tissue, mainly guided by the sole fat and bone conductivities variability.

These considerations, obtained with a systematic investigation of the exposure variability, further reinforce the results found in previous studies [10,19–22,50,51] which investigated, with different approaches, the effect of the dielectric properties variation on the pertinent electric quantity (electric field at low frequencies or Specific Absorption Rate at the radiofrequencies).

In summary, by integrating our results with the previous ones, it is shown that variations even higher than 1000% were neither clearly systematic nor sufficiently large to establish the variation in the dielectric properties as a decisive factor in the assessment of the global exposure of children, new-borns or foetuses.

## Supporting information

S1 File Data. (ZIP)
